# Up-regulation of HLA class-I antigen expression and antigen-specific CTL response in cervical cancer cells by the demethylating agent hydralazine and the histone deacetylase inhibitor valproic acid

**DOI:** 10.1186/1479-5876-4-55

**Published:** 2006-12-27

**Authors:** María de Lourdes Mora-García, Alfonso Duenas-González, Jorge Hernández-Montes, Erick De la Cruz-Hernández, Enrique Pérez-Cárdenas, Benny Weiss-Steider, Edelmiro Santiago-Osorio, Vianney Francisco Ortíz-Navarrete, Víctor Hugo Rosales, David Cantú, Marcela Lizano-Soberón, Martha Patricia Rojo-Aguilar, Alberto Monroy-García

**Affiliations:** 1 Laboratorio de Inmunobiología, Unidad de Investigación en Diferenciación Celular y Cáncer. FES-Zaragoza, UNAM, México; 2 Unidad de Investigación Biomédica en Cáncer, Instituto de Investigaciones Biomédicas, UNAM, Instituto Nacional de Cancerología, México; 3 Laboratorio de Biología Molecular del Cáncer, Unidad de Investigación en Diferenciación Celular y Cáncer, FES-Zaragoza, UNAM, México; 4 Departamento de Biomedicina, CINVESTAV, IPN, México; 5 Unidad de Reumatologia, IMSS, CMN SXXI, México; 6 Unidad de Investigación Médica en Enfermedades Oncológicas. IMSS, CMN SXXI, México; 7 Alumno del Doctorado en Ciencias Biológicas UNAM, México

## Abstract

**Background:**

DNA hypermethylation and histone deacetylation are epigenetic events that contribute to the absence or downregulated expression of different components of the tumor recognition complex. These events affect the processing and presentation of antigenic peptides to CTLs by HLA class-I molecules. In this work evaluated the effect of the DNA hypomethylating agent hydralazine and the histone deacetylase inhibitor valproic acid, on the expression of HLA class-I molecules and on the antigen-specific immune recognition of cervical cancer cells.

**Methods:**

Cell lines C33A (HPV-), CaSki (HPV-16+) and MS751 (HPV-18+) were treated with hydralazine and valproic acid to assess the expression of HLA class-I molecules by flow cytometry and RT-PCR. Promoter methylation of HLA class-I -A, -B and C, was also evaluated by Methylation-Specific PCR. Primary cervical tumors of four HLA-A*0201 allele patients were typed for HPV and their CTL's stimulated in vitro with the T2 cell line previously loaded with 50 μM of the HPV peptides. Cytotoxicity of stimulated CTL's was assayed against Caski and MS751 cells pre-treated with hydralazine and valproic acid.

**Results:**

Valproic acid and hydralazine/valproic acid up-regulated the constitutive HLA class-I expression as evaluated by flow cytometry and RT-PCR despite constitutive promoter demethylation at these loci. Hydralazine and valproic acid in combination but no IFN-gamma hyperacetylated histone H4 as evaluated by ChiP assay. The antigenic immune recognition of CaSki and MS751 cells by CTLs specific to HPV-16/18 E6 and E7-derived epitopes, was increased by VA and H/VA and the combination of H/VA/IFN-gamma.

**Conclusion:**

These results support the potential use of hydralazine and valproic acid as an adjuvant for immune intervention in cervical cancer patients whenever clinical protocols based on tumor antigen recognition is desirable, like in those cases where the application of E6 and E7 based therapeutic vaccines is used.

## Background

Aberrant gene transcription resulting from epigenetic changes, -namely DNA promoter hypermethylation and histone deacetylation- are frequent events in the molecular pathogenesis of malignant transformation [1,2]. Although cancer cells are less immunogenic than pathogens, the immune system is clearly capable of recognizing and eliminating tumor cells. However, tumors frequently interfere with immune response development and function through several mechanisms such as loss of antigen processing and presentation, the Fas counterattacking system, escaping from death receptor signaling, engaging in inhibition-blocking activation, suppression of antitumor responses by regulatory T cells, and tumor-induced immune suppression [3].

Current research demonstrates that epigenetic defects are involved in at least some mechanisms that preclude mounting a successful host-antitumor response, involving the HLA system, tumor-associated antigens, and accessory/co-stimulatory molecules [4,5]. Presentation of antigens within the context of HLA molecules is crucial both during T-cell priming and the effector phase of an adaptive immune response. Genetic alterations in antigen processing and presentation are commonly observed in malignancies, thus, complete HLA loss is a common event in several murine and human tumors [6,7]. DNA methylation participates in regulation of the expression of the three classes of human leukocyte antigen class-I antigens: HLA-A, HLA-B, and HLA-C, which are CpG-rich at their gene promoters [8]. Nie et al. showed down-regulation of HLA class-I antigens in esophageal carcinoma as a common mechanism for transcriptional inactivation caused primarily by DNA hypermethylation [9], as well as in melanoma, where 5-aza-2'-deoxycytidine significantly enhances the constitutive expression of HLA class-I antigens, of HLA-A1 and -A2 alleles, and of the co-stimulatory molecule, intercellular adhesion molecule-1, and lymphocyte function-associated antigen-3 [10]. Regarding HLA-Class-II, not only promoter hypermethylation [11,12] but also histone deacetylation have been found to account for the MHC-class-II-deficient phenotype of tumor cells. The treatment of CIITA- and MHC-class-II-deficient cells with the histone deacetylation agent trichostatin A results in the induction of CIITA, and resulting MHC class-II expression [13], in addition to the induction of expression of several other immunologically important molecules such as MHC class-I and CD40 [14].

Hydralazine (H), one of the first orally antihypertensive developed, is also a non-nucleoside DNA methylation inhibitor [15-17] whose demethylating and gene reactivating activity in tumors has also been demonstrated in a phase I trial in cervical cancer patients [18]. Valproic acid (VA) an 8-carbon, branched-chained fatty acid well-known as an effective antiepileptic drug causes hyperacetylation of the N-terminal tails of histones H3 and H4 *in vitro *and *in vivo *and inhibits HDAC activity [19,20]. Its ability to inhibit deacetylase activity in solid tumors has recently been demonstrated in cervical cancer patients [21], and when used in combination, these epigenetic agents show inhibitory growth effect in vitro and in vivo, and a synergistic effect upon global gene expression [22].

E6 and E7 proteins of high-risk human (HPV) types are thought to be the ideal sources of antigens for immunotherapy for cervical cancer because their persistence is necessary to maintain the transformed cell phenotype, moreover is known that E7 protein seems to induce protective cellular immunity in human premalignancy [23]. Since the majority of cervical cancer tumors show cells with a dysregulated expression of HLA class-I molecules on their surface that may affect the presentation of HPV-derived antigenic peptides to cytotoxic T cells [24,25], in this work we analyzed whether H and VA are able to up-regulate the expression of HLA class-I molecules on cervical cancer cell lines and whether they can promote the response to the presentation of known HPV16 E6 and E7-derived antigenic peptides to cytotoxic T cells derived from cervical cancer patients.

## Methods

### Cell lines and antibodies

C33A (HPV-), CaSki (HPV-16+) and MS751 (HPV-18+) human cervical carcinoma cell lines, which express in common the HLA-A2 allele on cell surface [26], as well as the SW480 colon carcinoma cell line, were maintained in RPMI-1640 medium (Gibco Laboratories, Grand Island, NY, USA) supplemented with 10% fetal bovine serum (HyClone, Inc. Laboratories Logan Utah. USA), penicillin G (100 IU/mL) (Lakeside, México), 100 μg/mL streptomycin sulphate (Sigma Chem. Co. St. Louis Mo, USA), and 2 mM L-glutamine (Sigma Chem. Co. St. Louis Mo, USA). Monoclonal antibodies were obtained from hybridomas supernatants and purified by elution in Protein-G sepharose (Sigma, Chem. Co. USA) columns: PA2.1 (Anti HLA-A2, -A28) MAb was purchased from American Type Culture Collection (Rockville, MD, USA) and the W6/32 MAb, which recognizes a conformational epitope on the intact heavy chain/β_2_microglobulin complex, was generously supplied by Dr. Gerd Moldenhauer of the German Cancer Research Center, Heilderberg, Germany.

### Hydralazine and valproic acid cell treatment

Cervical cancer cell lines were cultured in the presence of H, VA or both. Briefly: 5 × 10^5 ^cells were cultured in 6-well plates in the presence of 10μM of H (Sigma, Chem. Co. U.S.A) or 1 mM of VA (Sigma, Chem. Co. U.S.A) during 5 or 3 days respectively or with both drugs added together. On day 3 of cell culture, 2 mL of medium were removed and then added 2 mL of fresh complete medium containing the same concentration of drugs.

### Flow cytometry

To determine HLA class-I molecule expression on cell surface, 5 × 10^5 ^cells were treated with 10 μg/mL of each purified MAb, for 30 min. After washing the cells three times in 0.15 M NaCl-0.01 M phosphate buffer (pH 7.2)-2% fetal bovine serum (PBS-F), FITC-labeled goat anti-mouse Ig antibody (Gibco Co. U.S.A) was added to a dilution of 1:100 for 20 min on ice, followed by another two washes in PBS-F. Finally, the cells were resuspended in 0.5 mL of PBS-F and 1 μg/mL of Propidium Iodide (Sigma Chem. Co. USA) to discard cellular debris and then transferred to tubes. Cell samples were analyzed in a FACS-calibur flow cytometer (Becton Dickinson & Co. Mountain View, CA, USA). After gating out cell debris, 10 000 events were analyzed for their fluorescence intensity. In all experiments, the fluorescence intensity was determined at least three times where each of the 10, 000 events were gated. The staining with the FITC-labeled secondary antibody alone was considered as a negative control. The effect of human recombinant IFN-gamma on the HLA induction was determined in cell lines cultured with or without the presence of 200 U/mL of IFN-gamma (Genzyme Diagnostics, Cambridge, USA) for 48 hours [27]. The cells were then harvested and their HLA expression was determined as previously indicated.

### RT-PCR

Elution buffer. The PCR-amplification was carried out using the Advantage-GC Genomic PCR Kit (Clonetech) according to the manufacturer's instructions. PCR primer sequences for amplifying the human MHC class I promoter are enlisted in table 1. PCR products were separated on a 1.8% agarose gel and visualized by ethidium bromide staining.

**Table 1:** Primers and conditions used for PCR and RT-PCR analysis.

Primer set	Sense 5'-3'	Antisense 5'-3'	Size pb	Annealing
MHC-I promoter	ccagttcagggacagagattacggg	gagagggagaaaagaaactgcggag	280	60°C
HLA-A	gacagcgacgccgcgagcca	ggcagcgaccacagctccag	807	64°C
HLA-B	gacagcgacgccgcgagtcc	agtagcgaccacagctccga	907	60°C
HLA-C	gagatcacactgacctggca	gaacacagtcagtgtgggg	589	53°C
β-actin	gggtcagaaggattcctatg	ggtctcaaacatgatctggg	238	58°C
E7HPV16	cagctcagaggaggaggatg	gcccattaacaggtcttcca	166	60°C
E6HPV18	atgctgcatgccataaatgt	tgcccagctatgttgtgaaa	214	60°C
actin	acacctggacctggactcac	gctcttggctcctttgtcac	231	60°C
MY09	cgtccmarrggawactgatc			45°C
MY11		gcmcagggwcataayaatgg	450	

### Stabilization assays of the HLA-A2 allele with HPV E6 and E7-derived peptides

The antigenic peptides TLGIVCPIC and YMLDLQPETT derived from the E7 HPV-16 protein and the KLPDLCTEL derived from the E6 HPV-18 protein that specifically bind to HLA-A2 allele [28-30], were synthesized by Invitrogen, USA, dissolved in phosphate buffered saline and stored at -70°C before use. The peptide GILGFVFTL derived from the M Influenza-A protein, was used as positive control for binding assay to the empty HLA-A2 molecule. Lymphoblastic T2 cell line, which express empty HLA-A*0201 molecules on its cell surface [31], was used to test the affinity of different concentrations (12.5–100 μM) of each synthetic peptide after incubation overnight at 37°C in the presence of 5 μg/mL of β_2_-microglobulin (Sigma, USA).

### Clinical samples

Biopsies were taken from areas with visible macroscopic cervical tumor using a sterile biopsy punch. Part of the biopsy was sent to the Institution's Pathology Department for routine hematoxilin & eosin diagnosis. The remaining biopsy specimen was immediately frozen at -20°C for HPV typing. In addition, 20 mL of peripheral blood were drawn from the arm by venipuncture to obtain the mononuclear cell fraction in order to stimulate the cytotoxic T lymphocytes. The protocol was approved by the Institutional Regulatory Boards and patients signed an informed consent before blood and sample tissues were taken.

### HPV typing

The MY09 and MY11 L1 consensus primers (Table 1) that recognize a conserved region in the L1 open reading frame, producing a fragment of 450 bp, were used to examine the presence of HPV DNA in the genomic DNA of each β-globin positive tumor sample.

The reaction was carried out in a final volume of 25 μL containing 400 ng of DNA, 1.5 mM MgCl_2_, 200 μM of dNTPs, 0.4 μM of each of the primers and 1U of Taq DNA polymerase (GIBCO BRL, Grand Island, NY). The positive control consisted of DNA from CaSki and MS751 cell lines, which contain the HPV type 16 and 18 genome respectively. The conditions of amplification were as follows: Denaturing at 94°C for 15 sec, primer annealing at 58°C for 30 sec and extension at 72°C for 1 min, for a total of 35 cycles, the final cycle included an incubation at 72°C for 10 min. 7 μL of amplification product were electrophoresed in 1.5% agarose containing 0.5 μg/mL of ethidium bromide and visualized by UV light. Positive MY09/MY11 products were digested with Bam HI and Rsal restriction enzymes (Biolabs, USA). The restricted samples were electrophoresed on a 3% agarose gel stained with ethidium bromide. The restriction fragment length polymorphism (RFLP's) obtained were compared with that reported by Bernard [32].

### In vitro induction of CTL responses

To stimulate CTLs, we used a method previously reported [33]. Briefly: 4 × 10^6 ^Peripheral Blood Lymphocytes (PBLs) were resuspend in 1 mL of complete medium consisting of Iscove's Modified Dulbecco's Medium (Gibco BRL, USA) supplemented with 10% heat-inactivated FBS, 100 IU/mL penicillin, 4 mM L-glutamine, 1 mM sodium pyruvate and 20 μM 2-mercaptoethanol, and incubated with 10 μM of peptide in 24-wells plates. On day 3, the wells were topped up with 1 mL of complete medium containing recombinant human IL-2 (final concentration 10 IU/mL, R&D). On day 7 and weekly thereafter, the cells were restimulated as follows: (a) we used T2 cell line as antigen presenting cell; 1 × 10^5 ^T2 cells previously loaded with 50 μM of the peptides in the presence of β_2_-microglobulin and fixed with 0.1% glutaraldeyde in PBS, were incubated with 5 × 10^5 ^T cells; (b) 1 × 10^6 ^responder T cells were added in 1 mL of complete medium; and (c) cells were topped up 2 days later with 1 mL of complete medium containing hrIL-2 and hrIL-15 at final concentration of 10 IU/mL and 15 ng/mL respectively. Cytotoxicity assays were performed on day 21.

### Cytotoxicity assays

Cervical cancer cell lines alone or pretreated with H, VA, both, IFN-gamma or H/VA/IFN-gamma as indicated, were used as target cells after labeled with ^51^Cr (Amersham, USA) for 1 h. Different numbers of effector cells in 50 μL of complete medium were incubated and then 2.5 × 10^3 ^^51^Cr-labeled target cells were added to triplicate wells of 96-well plates in final volume of 200 μL. After 4 h at 37°C, 100 μL of supernatant were harvested and transferred to counting vials and measured on a γ-counter (Packard, U.S.A). For each pretreated cell group, ^51^Cr-labeled cells incubated with 5% SDS or medium alone were used to determine maximum and spontaneous releases. Spontaneous release was usually less than 10% and never exceeded 15%. The percentage of specific lysis of each well was calculated as: (experimental release - spontaneous release)/(maximal release - spontaneous release) × 100.

### Statistical analysis

All numerical data were expressed as average of values obtained ± standard deviation (SD) of experiments made by triplicate. Comparisons were evaluated by unpaired *t *test. A *p *value <0.05 was considered significant.

## Results

### Hydralazine and valproic acid effects upon expression of HLA class-I molecules at the cell membrane

**Figure 1:**
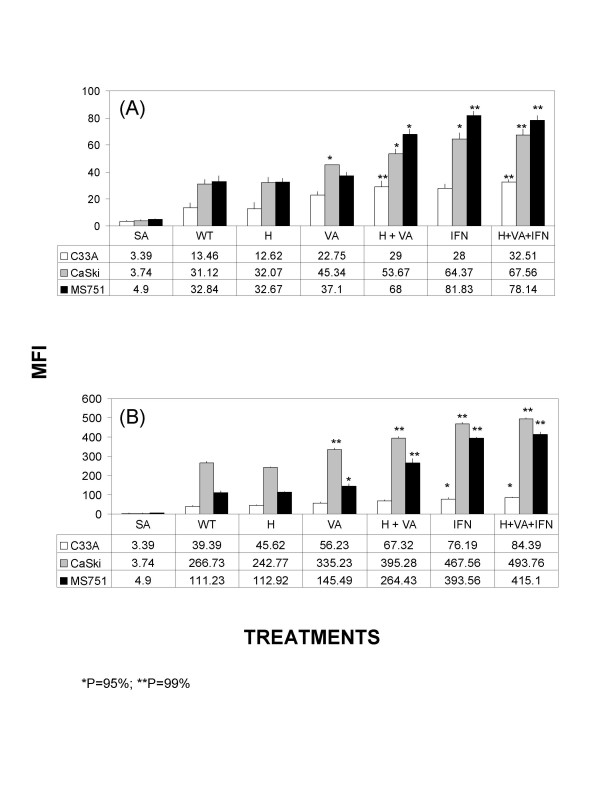
**Effect of H and VA on the expression of HLA class-I antigens on cervical cancer cells**. C33, CaSki and MS751 cells were incubated in the presence of H and VA and either HLA-A2 (**A**) or total HLA class-I (**B**) expression was determined by flow cytometry as indicated in materials and methods. The mean of 10 000 events is depicted in each cell treatment. Significant differences, * and ** were obtained in comparison to untreated cells (wt).

To determine whether these epigenetic agents enhance the constitutive expression of HLA class-I molecules, the expression analysis of the HLA-A2 allele and total HLA class-I molecules was carried out by using PA2.1 and W6/32 MAbs. The results showed that HLA-A2 allele expression level was unchanged in the C33A cells by hydralazine alone whereas VA, H/VA, IFN-γ and H/VA/IFN-γ increased one-fold its expression. Regarding total class-I molecules, the increasing effect was unexistent except for a small increase by IFN-γ and H/VA/IFN-γ. In CasKi cells, a similar pattern of increased expression was observed in HLA-A2 allele and total HLA class-I molecules expression by these drugs and combinations except for hydralazine alone treatment. In particular for total HLA class-I, it seems there was a summatory effect among the three drugs, H/VA/IFN-γ. Of note the effect seen on CasKi cells in HLA-A2 allele and total HLA class-I molecules by these drugs and combinations was almost identical in the MS751 cells. Statistical significance among cell lines and treatments in comparison to untreated (wt) are shown (Figure 1).

### Transcriptional effect of hydralazine and valproic acid upon expression of HLA class-I molecules

**Figure 2:**
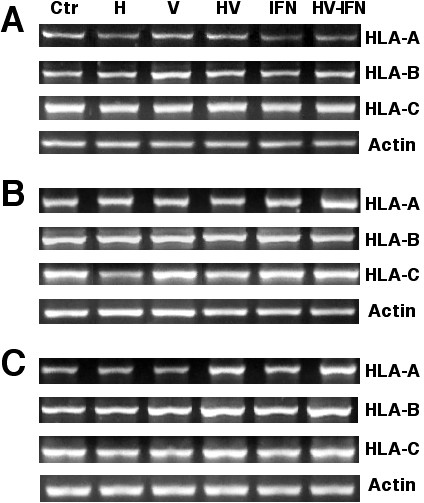
**Transcriptional effect of hydralazine and valproic acid upon expression of HLA class-I molecules**. Total RNA was extracted from C33A, CaSki and MS751 cells treated with H, VA or H/VA. RT-PCR analysis was performed using HLA-A, -B or -C and β-actin primer pairs (Table 1). PCR products were then separated on a 3% agarose gel. One hundred-bp markers (MW) were run in the flanking lanes of each gel. Human recombinant IFN-gamma was used as a positive inducer of the HLA class I expression. RNA of the peripheral blood lymphocytes (PBL) from a normal donor, was used as a positive control. **A **is C33A, **B **is Caski and **C **corresponds to MS751 cell lines. Changes in expression as determined by densitometric analysis according to treatments are described in results.

To investigate whether the up-regulating effects of these drugs of HLA class-I molecules as shown by flow cytometry could be mediated by increased transcription, treated cell lines were analyzed by RT-PCR. Figure 2 shows that C33A cells despite had no increase in transcript levels for the HLA-A and -C genes with any combination of treatments, HLA-B gene showed a 0.35, 0.29, 0.21 and 0.42 fold-increase in band intensities with H, VA, H/VA and H/VA/IFN-gamma respectively. In CasKi cells where HLA-A2 was most increased by IFN-gamma and H/VA/IFN-gamma the fold increases in band intensity were 0.13 and 0.91 respectively. HLA-B was also increased 0.12, 0.43 and 0.28-fold with H/VA, IFN-gamma and H/VA/IFN-gamma respectively. In HLA-C, an increase of 0.25 and 1.4-fold were observed with IFN-gamma and H/VA/IFN-gamma. The MS751 cell line showed increases of the same magnitude in band intensities with all the combinations except for H alone. In particular for HLA-A gene, the triple combination of H/VA/IFN-gamma led to a 1.29-fold increase.

### Methylation and acetylation of HLA Class-I genes

**Figure 3:**
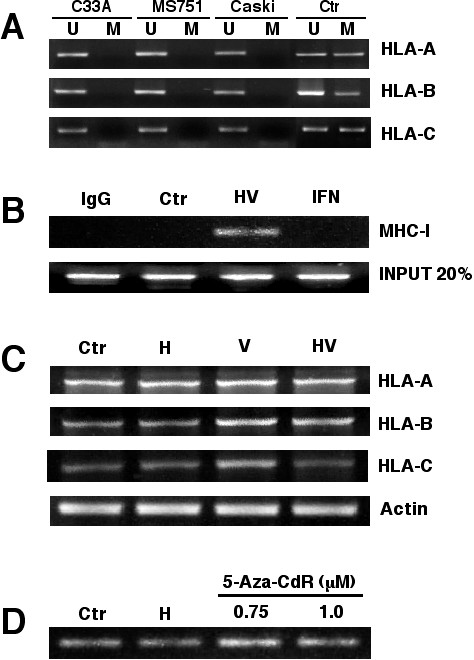
**Methylation and acetylation of HLA Class-I genes**. **A**. HLA-A, -B anc -C gene promoters were analyzed by MSP in the cervical cancer cell lines. The control for unmethylated reaction is modified DNA from normal lymphocytes and for methylated is the same DNA but methylated in vitro. **B**. Chromatin immunoprecipitation assay with an anti-H4 acetylated antibody in Caski cells. Treatment with H/VA but no IFN-gamma led to H4 hyperacetylation. **C**. RT-PCR analysis of HLA-A, -B and -C, after treatment with H, H/VA and H/VA in SW480 colon carcinoma cell line and D. MSP (only with methylated primers shown) of the HLA-B promoter in SW480 cells indicating the lack of demethylation by H and 5-aza-2'-deoxycytidine.

Previous studies have demonstrated that epigenetic mechanisms are main regulators of the expression of this class of molecules and that both DNA methylation and HDAC inhibitors demethylate and reactivate their expression. To investigate this issue, we determined by methylation-specific PCR the methylation status at the gene promoter of HLA-A, -B and -C genes in C33A, CasKi and MS751 cell lines. As shown in figure 3a, there was complete demethylation at these three promoters in all the cell lines investigated. The absence of gene promoter at these genes prompted us to analyze whether histone acetylation could be responsible for the increase expression seen by the epigenetic drugs used. As shown in figure 3b, chromatin immunoprecipitation assay showed that the combination of H/VA but no IFN-γ led to H4 hyperacetylation at the HLA-class-I promoter. Because hydralazine can be considered as a weak DNA methylation inhibitor and it has been reported that 5-aza-2'-deoxycytidine does demethylate the HLA-B promoter in the KYSE esophageal carcinoma cell line, we searched the expression of HLA-A, -B and -C genes and the promoter methylation status in several cell lines. We found that the SW480 colon carcinoma cell line had methylated the HLA-B locus. When this cell line was treated with H, VA and H/VA, like to that observed for cervical cancer cell lines, VA and H/VA led to small but clear increase in expression level of the three loci, however, neither H nor 5-aza'-2'-deoxycytidine demethylated the HLA-B locus (Figure 3c and 3d).

### Treatment with VA and H/VA increase the immune recognition of cervical cancer cells by CTLs stimulated with HPV-16 and HPV-18 E6/E7 derived epitopes

**Figure 4:**
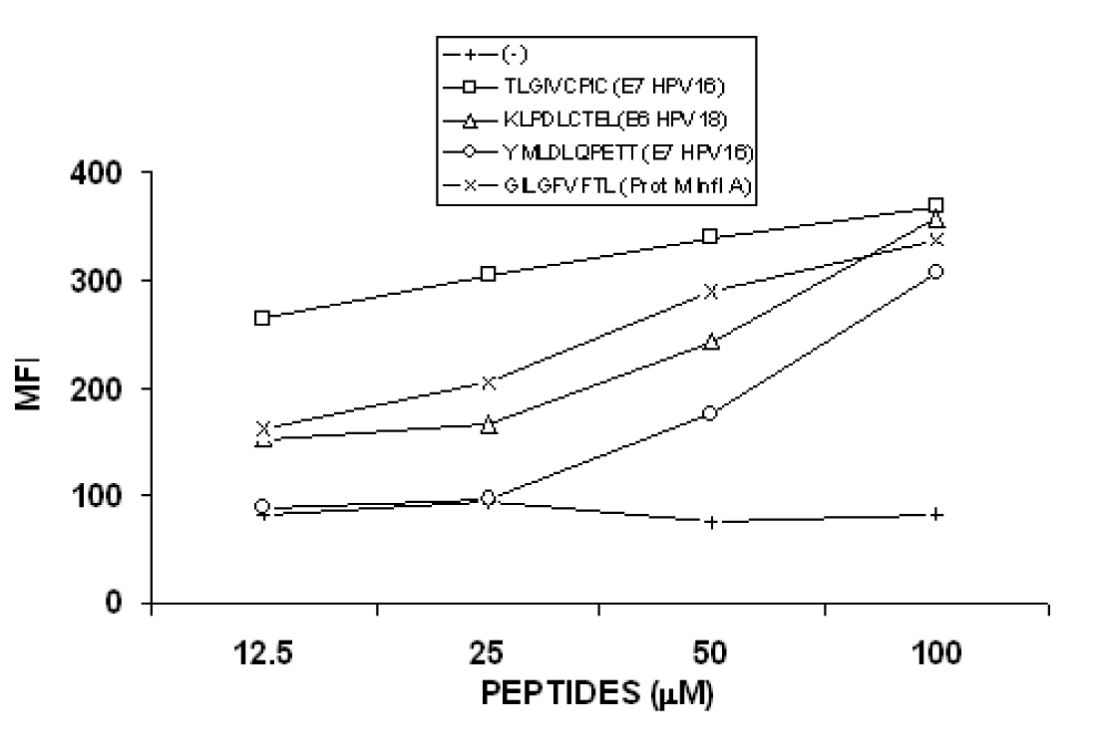
**HLA-A2 allele stabilization with peptides derived from the E6 and E7 proteins**. The lymphoblastic T2 cell line, which express empty HLA-A*0201 molecules on their cell surface, was loaded with different concentrations of the peptides: TLGIVCPIC and YMLDLQPETT derived from the E7 HPV-16 protein and KLPDLCTEL of the E6 HPV-18 protein. The peptide GILGFVFTL derived from the M Influenza-A protein, was used as positive control for binding assay. After incubation overnight with the peptides, conformational expression of the HLA-A2 allele was determined by flow cytometry using the PA2.1 MAb. MFI, mean fluorescence intensity.

**Figure 5:**
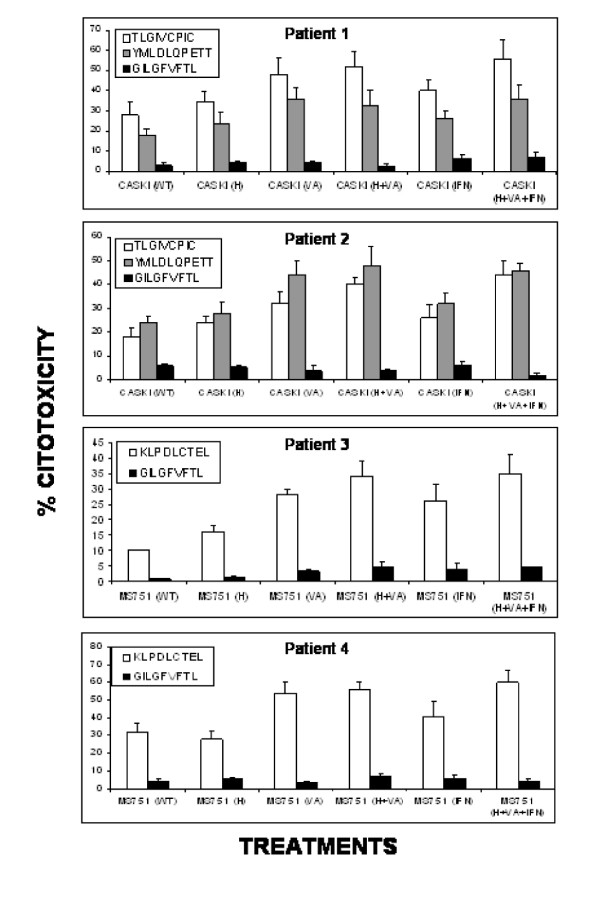
**CTL recognition of CaSki and MS751(HPV-18+) cell lines treated with H and VA**. T lymphocytes derived from patients with squamous cervical cancer, positives either for HPV-16 or -18 infection (Table 2) were stimulated with the peptides TLGIVCPIC and YMLDLQPETT of the E7 HPV-16 protein and the peptide KLPDLCTEL of the E6 HPV-18 protein respectively and their cytolytic activity was tested by a standard 4-h 51Cr release assay against CaSki(HPV-16+) or MS751(HPV-18+) without treatment (WT) or previously treated with H, VA or H plus VA as indicated.

To analyze whether the treatment of cervical cancer cells with hydralazine and valproic acid is also able to increase their immune recognition, T lymphocytes derived from cervical cancer patients with HPV-16 or HPV-18 infection and with the HLA-A2 allele in their HLA Class-I haplotype, were stimulated with three known E6 and E7 HPV derived antigenic peptides, that specifically bind to the HLA-A*0201 allele [28-30]. Two of the peptides TLGIVCPIC and YMLDLQPETT were derived from the E7 HPV-16 protein and the other one KLPDLCTEL derived from the E6 HPV-18 protein. We also used the lymphoblastic T2 cell line to stimulate T lymphocytes contained in PBL's from patients with cervical cancer. Due to the fact that the T2 cells express empty HLA-A2 molecules on their cell surface [31], we previously performed peptide binding assays to analyze the binding affinities for these peptides. Using 50–100 μM of these three peptides, we observed an efficient stabilization of the HLA-A2 allele on T2 cells similar to the one obtained with the control peptide GILGFVFTL derived from the protein M of the influenza-A and with high binding affinity to the HLA-A2 allele (Figure 4). The T lymphocytes used were obtained from four patients with cervical squamous cell carcinoma. Two of those with HPV-16 infection (Patients 1, 2) and two with HPV-18 infection (Patients 3 and 4) all positive for the HLA-A*0201 allele (Table 2). The lymphocytes were stimulated during three rounds with the T2 cells loaded with the three antigenic peptides and then challenged against CaSki or MS751 cells that were previously treated with H, VA, H/VA, IFN-gamma and H/VA/IFN-gamma. We observed as expected, that T lymphocytes from the patients 1 and 2, that were positive for HPV-16 infection and stimulated with T2 cells loaded with the peptides TLGIVCPIC and YMLDLQPETT were able to lyse CaSki cells (Figure 5) and that this cytotoxicity mainly increased when the cells were previously treated with VA, H/VA, IFN-gamma and H/VA/IFN-gamma. Of note cytotoxicity was at least if not higher with any of these combinations as compared to IFN-gamma alone. On the other hand the T lymphocytes derived from the two patients with HPV-18 infection (Patients 3 and 4) and stimulated with the T2 cell line loaded with the peptide KLPDLCTEL, were able to lyse MS751 cells. In patient 3, the higher cytotoxicity was found with VA, H/VA and H/VA/IFN-gamma whereas in patient 4, the cytotoxic effect on cells treated with H/VA, IFN-γ and H/VA/IFN-gamma was essentially of the same magnitud but higher than IFN-gamma alone. (Figure 5). In all experiments T lymphocytes stimulated with the E6 and E7 epitopes were always capable to lyse the T2 cell line loaded with the proper antigenic peptide (data not shown). Moreover, we observed a very low T cell cytotoxic activity on CaSki and MS751 cells when T lymphocytes previously stimulated with the control peptide GILGFVFTL were challenged against these cells

**Table 2:** HLA class-I alleles of the patients with cervical cancer and their tumor characteristics.

Patient	Diagnostic	Clinical stage (*)	HLA class-I haplotype	HPV type
1	SCC	IIIB	**A2**, B53, Cw4 A68, B48, Cw3	16
2	SCC	IB1	**A2**, B39, Cw3 **A2**, B60, Cw3	16
3	SCC	IIA	**A2**, B38(16), Cw3 A2, B60(40), Cw3	18
4	SCC	IIIB	**A2**, B35, Cw4 A3, B41, Cw7	18

(*) FIGO clinical staging system.

### Hydralazine and valproic acid effects upon expression of HPV viral oncogenes

**Figure 6:**
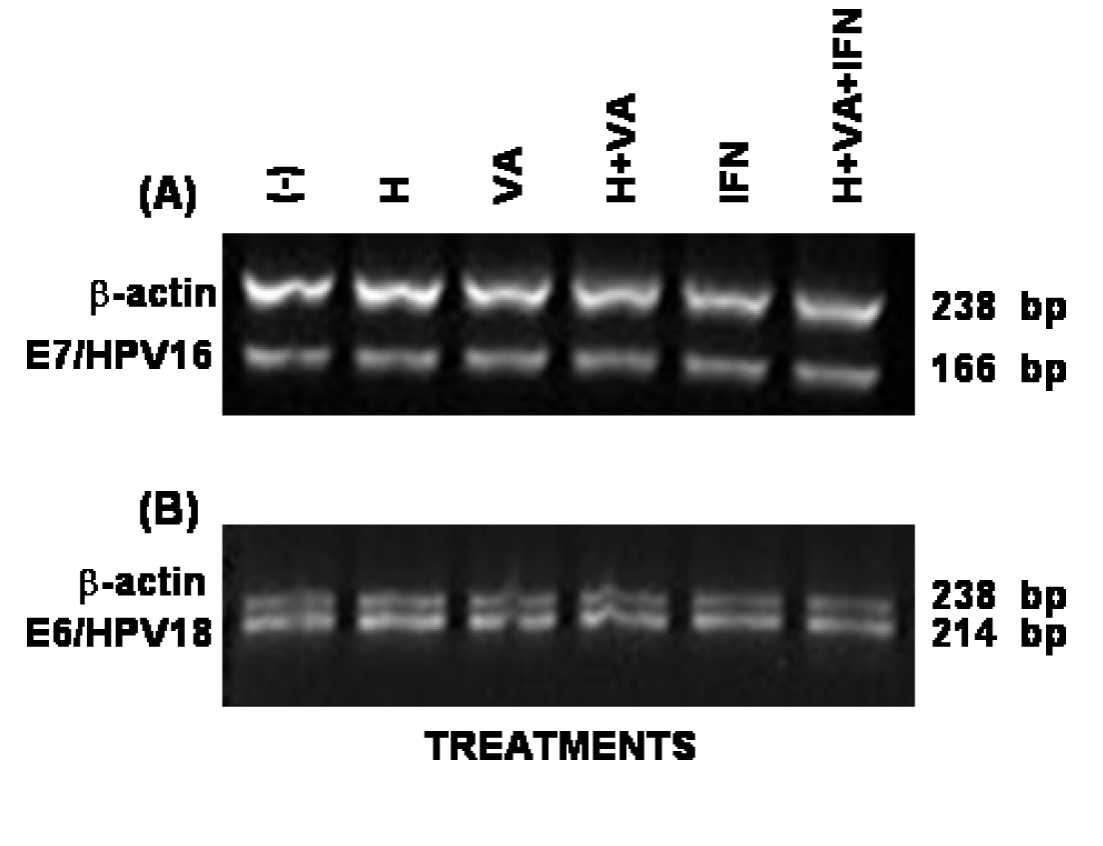
**HPV-16 E7 and HPV-18 E6 gene expression in CaSki and MS751 cell lines treated with H and VA**. Total RNA was extracted from CaSki and MS751 cell lines treated with either H, VA or H plus VA and then RT-PCR analysis for the HPV-16 E7 and HPV-18 E6 gene expression in CaSki and MS751 was respectively performed using E7 and E6 primer pairs (Table 1). The effect of human recombinant IFN-γ and the sum of H/VA/IFN-γ was also tested.

To investigate whether these epigenetic agents modulate the expression of E6 and E7 genes in the Caski and MS751 cell lines, the expression of these genes was analyzed by RT-PCR. The results show that neither E7 transcript of the HPV-16 nor E6 transcript of the HPV-18 were changed by drug treatment suggesting that the enhanced immune recognition of CaSki and MS751 cells by CTLs derived from cervical cancer patients can be mainly due to the increased presentation of antigenic peptides by the increased expression of HLA class-I molecules on cell surface rather than by an increase in E6 or E7 peptides (figure 6).

## Discussion

In this work we present evidence that the antigen-specific recognition of cervical cancer cells by cytotoxic T lymphocytes, is enhanced by the treatment of the cancer cells with the histone deacetylase inhibitor valproic acid alone or in combination with the DNA methylation inhibitor hydralazine. This effect can be attributed to the improved antigen presentation on the cell surface as a result of at least partially from increased transcription of HLA class-I molecules in treated cells. Although up-regulation of these class-I molecules has already been observed to occur after cells are treated with a demethylating agent [10] or with a histone deacetylase inhibitor [14] our results demonstrate that in some cell lines and patients the up-regulation is higher with the combination as compared to the individual effect of these drugs. A synegistic effect upon cancer testis antigens NY-ESO-1 and MAGE-A3 expression has been observed with 5-aza-2'-deoxycytidine and depsipeptide [34] but not upon HLA class-I molecules. Here we demonstrate that hydralazine and valproic acid synergize in this regard. This observation is supported by our previous study in which SW480 cells showed up-regulation of major histocompatibility complex, class-I-related only with the combined treatment but no with hydralazine or valproic acid alone [22]. Interestingly, in CasKi and MS751 cells H/V slightly increase the up-regulation when added to IFN-γ, as compared to IFN-γ alone, a potent and well-known inducer of HLA-class-I expression [33].

Previous studies have reported that the *de novo *expression of HLA class-I antigens induced by 5-aza-2'-deoxycytidine seems to be a sporadic phenomenon, since it was observed only in one melanoma cell line [35] and in a human esophageal cell carcinoma cell line [9], but not in a panel of HLA class-I-negative or HLA-A2-negative melanoma cells [36]. Consistent with an up-regulatory and not with a the *de novo *re-expression effect we also observed that these three cervical cell lines showed basal mRNA expression of HLA-A, -B and -C loci as well as constitutive expression of antigen processing components such as LMP-2, LMP-7, LMP-10 catalytic subunits of the proteasome and the transporters TAP-1 and TAP-2 (data not shown). It was of interest the observation that the effect of hydralazine was consistent regarding the lack of effect in the expression of HLA class-I molecules as in the cervical cancer cell lines tested the HLA-A, -B and -C promoters were unmethylated. Interestingly, despite 5-aza-2'-deoxycytidine has shown the ability to demethylate HLA-B locus in a an esophageal carcinoma cell line, both hydralazine and the nucleoside analog which is the prototype demethylating agent failed to demethylate the promoter in the SW480 cell line despite 5-aza-2'-deoxycytidine increased gene expression. This clearly indicates that at least in this model, chromatin remodelling by histone acetylation predominates over methylation regarding the regulation of gene expression.

Besides the well demonstrated antitumor effects of epigenetic therapies achieved by restoring the expression of key genes responsible of the malignant phenotype [2], the restoration of the defective expression of distinct components of the "tumor recognition complex" through epigenetic targeting of cancer cells results in their efficient recognition and lysis by antigen-specific CTL. In fact, *de novo *expression of selected cancer tumor antigens induced by 5-aza-2'-deoxycytidine allowed specific CTL recognition of melanoma, lung cancer, esophageal cancer, mesothelioma, renal cell carcinoma and sarcoma cells [34,37-39]. Furthermore, the up-regulated expression of HLA class-I antigens and allospecificities observed in melanoma cell lines after exposure to 5-aza-2'-deoxycytidine resulted in their increased recognition by a gp 100-specific HLA-A2-restricted CTL clone [40].

Accordingly, the treatment of Caski and MS751 cell lines with H, VA, IFN-γ or H/VA/IFN-γ enhanced their specific recognition by the patients CTL's raised against specific related peptides (TLGIVCPIC and YMLDLQPETT) of the E7 HPV-16 protein and (KLPDLCTEL) of E6 HPV-18 but no against the control peptide. Interestingly, the cytotoxicity was higher with VA or H/VA and the combination of H/VA/IFN IFN-gamma suggesting that in our system chromatin remodeling by histone HA acetylation could be the key determinant for the enhanced specific recognition of cancer cells by CTLs. In fact, whereas histone acetyltransferases promote CIITA function in transactivation of MHC genes, histone deacetylases [41] interfere with this CIITA function following IFN-gamma induction. Of note, the observed cytotoxicity was higher with VA than with IFN-gamma. It is known that histone deacetylation impairs the transactivation of MHC genes by IFN-gamma, accordingly, in CaSki and MS751 cells, it seems that H/VA slightly increase the expression.

The role of HPV genome DNA hypermetylation is currently being studied. Existing information suggests that methylation status of viral oncogenes in lesions is perhaps solely the result of their transcriptional activity level and not a causal event for neoplastic progression [42]. Here we also found no changes of HPV-16 E7 on CaSki cells and HPV-18 E6 on MS751. This result is in line with observations that HLA-A*0201-restricted CTL clones against HPV-16 E6_29–38 _that recognize HPV-16 E6 antigens transfected into B lymphoblastoid cells are unable to recognize HLA-A*0201(+) HPV16 E6(+) cervical carcinoma cell lines even when the level of endogenous HPV-16 E6 in these cells was increased by transfection. In addition, the defect in presentation of HPV16 E6 [25-27,40-43] correlates with low level expression of HLA class-I, proteasome subunits low molecular mass protein 2 and 7, and the transporter proteins TAP1 and TAP2 in the cervical carcinoma cell lines, suggesting that presentation of the HPV16 E6 epitope in cervical carcinoma cell lines is limited by mechanisms other than the level of HPV16 E6_29–38 _epitope availability [43].

To the best of our knowledge this is the first study showing an up-regulated HLA class-I expression and antigen-specific CTL response in cervical cancer cells following the use of hydralazine and valproic acid. It will be of interest to investigate whether epitopes derived from proteins whose genes have been reactivated by hydralazine and valproic acid, different from those derived from HPV oncogenic proteins can be specific targets for CTL immune recognition. In fact, ongoing laboratory data from our group demonstrate that these drugs have the ability to increase the expression of tumor associated antigens such as MAGE and GAGE families in cervical cancer cell lines (data not shown). In addition, this combination of epigenetic agents may also help to avoid immune evasion strategy of tumors by up-regulating the expression of the major histocompatibility complex, class-I-related, a powerful NKG2D ligand for NK cell-mediated antitumor immunity [44] as we have observed it in a colon carcinoma cell line treated with hydralazine and valproate [22].

## Conclusion

The development of more effective immunotherapy strategies calls for a better understanding of among other, the mechanisms underlying immune evasion by tumors cells [45]. The results of this study suggest that use of epigenetic drugs such as hydralazine and valproic acid could improve immune interventions in clinical trials based on E6 and E7 peptides, due to their up-regulating effect on HLA class-I molecules.

## Competing interests

The author(s) declare that they have no competing interests.

## Authors' contributions

MLMG, performed cell culture and H/VA treatment of the cells as well as HPV typing; DC, participated the collection and storing of clinical samples; MLS performed RT-PCR analysis; ESO performed T lymphocyte activation; JHM participated in HLA typing and cytotoxicity assays; VHR and MPRA participated in FACS analysis; EPC and ESH performed methylation and chromatin immunoprecipitation assays; ADG, VFON and BWS participated in discussion of results and revision of the manuscript; AMG conceived of the study and wrote the manuscript.
